# Couple Relationship Quality and the Infant Home Language Environment: Gender-Specific Findings

**DOI:** 10.1037/fam0000590

**Published:** 2019-08-22

**Authors:** Elian Fink, Wendy V. Browne, Isla Kirk, Claire Hughes

**Affiliations:** 1 Centre for Family Research and Faculty of Education, University of Cambridge; 2 Centre for Family Research, University of Cambridge

**Keywords:** speech, vocalizations, couple relationship quality, infant

## Abstract

Couple relationship quality is known to drop significantly across the transition to parenthood (Ahlborg & Strandmark, 2001; Doss, Rhoades, Stanley, & Markman, 2009), yet individual differences in the amount of parent-to-infant talk have rarely been studied in relation to variation in couple relationship quality. Addressing this gap, the current study of 93 first-time parents with 4-month-old infants included multimeasure reports of couple relationship quality from both mothers and fathers and examined associations between couple relationship quality and the home language environment, assessed via the Language Environment Analysis (LENA), when infants were approximately 7 months old. LENA consists of a wearable talk pedometer that records a full day of naturalistic parent-infant talk and is coupled to software that provides automated analysis. Given the covariation between depression and both couple relationship quality and parental infant-directed talk, both maternal and paternal depression were controlled for in all analyses. Results showed that, for mothers of sons, frequency of infant-directed talk was inversely related to couple relationship quality. Consistent with family systems theory, this finding provides partial support for the compensation hypothesis. However, variation in couple relationship quality was unrelated to infant-directed speech in fathers or in mothers of daughters. Together, these findings demonstrate that the gender composition of the parent-infant dyads plays a moderating role on the association between couple relationship quality and parent-infant talk.

The early home language environment is crucial for children’s development, especially regarding verbal-based skills (e.g., Hart & Risley, 1995; Rowe, 2008). Given that the transition to parenthood adversely affects both couple relationship quality and mental health (Ahlborg & Strandmark, 2001; Doss, Rhoades, Stanley, & Markman, 2009; Gjerdingen & Center, 2003; Parfitt & Ayers, 2014) and the known negative impact of depression and anxiety on infant directed speech (e.g., Davis, Davis, Freed, & Clark, 2011; Jacob & Johnson, 1997; McLearn, Minkovitz, Strobino, Marks, & Hou, 2006; Paulson, Dauber, & Leiferman, 2006; Sohr-Preston & Scaramella, 2006), it is striking that the association between couple relationship quality and infant directed speech has yet to be investigated. By harnessing Language Environment Analysis (LENA) technology to collect a full day of naturalistic mother and father infant-directed talk, ratings from both parents on a multimeasure indicator of couple relationship quality (including couple satisfaction, conflict, and support) and controlling for both maternal and paternal depression at 4 months postpartum, the current study aimed to examine the unique association of couple relationship quality on infants’ home language environment.

## The Importance of Parental Speech

Alongside the complexity of parent-child contingent conversation, numerous studies have demonstrated that the overall quantity of child-directed parental speech predicts children’s later language skills (e.g., Hart & Risley, 1995; Rowe, 2008; Weisleder & Fernald, 2013; Zimmerman et al., 2009), which in turn is important for the development of early social skills (Cole, Armstrong, & Pemberton, 2010; Gallagher, 1993; Guellai & Streri, 2011).

Unfortunately, existing studies of conversational contributions to children’s cognitive development rely heavily on brief home- or laboratory-based observations of parent-child interactions (e.g., Hirsh-Pasek et al., 2015; Huttenlocher, Waterfall, Vasilyeva, Vevea, & Hedges, 2010; Rowe, 2008, 2012; Weizman & Snow, 2001). Given the limitations associated with this approach (e.g., lack of representativeness/ecological validity, poor sensitivity to individual differences), there is obvious interest in harnessing technological advances to assess child-directed parent language over longer periods of time. The LENA device is a digital audio recorder that provides up to 16 hr of naturalistic data on the infant’s linguistic environment and is paired with software to estimate the total number of infant-directed adult words (distinguishing between male and female voices) and adult-infant conversational turns.

Applying LENA technology to a sample of 275 families with children aged from 2 to 48 months of age, Zimmerman et al. (2009) found that the frequency of adult-child conversations based on more than a full day of interactions each month for 6 months were robustly associated with the child’s concurrent language ability. Reflecting the increased granularity of measures available using the LENA software, LENA has enhanced our understanding of the importance of the specific features of parent-child interactions, such as contingent conversation (e.g., Romeo et al., 2018), that support the development of children’s language skills. Automation also dramatically reduces the work-hours needed to code a full day of naturalistic parent-child interactions, thereby enabling studies to recruit larger sample sizes (Wang et al., 2017).

Recruiting larger samples also enables researchers to investigate how the gender composition of the parent-child dyad may contribute to variation in parental talk. Although existing research has focused heavily on mothers, some differences between mother and father talk have been identified. For example, compared with mother talk, father talk has been reported to contain more problem-solving–related comments but less likely to be spoken from the point of view of the infant (Lundy, 2003). Furthermore, when compared with mothers, fathers tend to be more animated, active, and reactive but less infant-directed (Kokkinaki, 2018). Infant gender also appears to be important. For example, a study using LENA found that mothers with daughters responded to infant-initiated vocalizations more often than mothers with sons, whereas fathers with sons made marginally more responses than did fathers with daughters in the first 7 months of life (Johnson, Caskey, Rand, Tucker, & Vohr, 2014).

## Couple Relationship Quality and Family Interactions

The transition to parenthood has been conceptualized as a crisis in the couple dynamic; not only do the mother and father have their own newfound pressures as parents, but as a couple, routine and balance is interrupted (Epifanio, Genna, De Luca, Roccella, & La Grutta, 2015). Reduced couple satisfaction, increased couple conflict, and lower levels of partner support have each been observed in first-time mothers and fathers (Bost, Cox, Burchinal, & Payne, 2002; Crohan, 1996; Doss et al., 2009). Relatedly, poor relationship quality is linked to poorer mental health in parents (e.g., Figueiredo et al., 2018). There is also substantial evidence to demonstrate that the degree of support and conflict in the parental relationship influences the parent-child relationship and child outcomes (e.g., Belsky, Youngblade, Rovine, & Volling, 1991; Grych, 2002; Harold & Sellers, 2018), with parental talk to their child likely playing an important role in the association between couple relationship quality and child outcomes (Pancsofar, Vernon-Feagans, Odom, & Roe, 2008; Pratt, Kerig, Cowan, & Cowan, 1992). For example, parents of 12-month-old infants with a positive couple relationship (i.e., high levels of marital love) were found to use more varied vocabulary than those families with lower levels of couple relationship quality (Pancsofar et al., 2008), with varied vocabulary use during infancy and toddlerhood in turn associated with greater child vocabulary development (Rowe, 2012).

To understand how couple relationship quality might influence parent-child interactions, family systems theory (e.g., Erel & Burman, 1995) provides a useful perspective that highlights two competing processes. In the first of these, known as spillover, couple relationship quality transfers directly onto the parent-child relationship, such that any negativity generated from the interparental relationship is associated with negative parenting behaviors and more dysfunctional parent-child interactions (Erel & Burman, 1995). In the second process, known as compensation, parents counteract experiences of poor relationship quality by dedicating more time and effort into their relationship with their child (Engfer, 1988).

Meta-analytic reviews suggest that spillover is more commonly observed than compensation (e.g., Erel & Burman, 1995; Krishnakumar & Buehler, 2000), but studies involving mothers and fathers highlight gender-specific effects. In particular, spillover appears stronger in fathers than mothers (Kerig, Cowan, & Cowan, 1993; Parke, 2002). For example, research has shown that poor couple relationship quality has greater adverse effects on paternal than maternal parenting behaviors (Coiro & Emery, 1998). Conversely, mothers appear more likely than fathers to display compensation effects. For example, in a study of 203 families with young adolescent children, Kouros, Papp, Goeke-Morey, and Cummings (2014) found that poor couple relationship quality was related to better mother-child relationship quality. Importantly, spillover and compensation effects are not mutually exclusive. Moreover, research findings suggest a complex interplay between parent/child gender and other family stressors, including parental depression (e.g., Nelson, O’Brien, Blankson, Calkins, & Keane, 2009). For example, child gender has been shown to moderate the association between parental conflict and parenting behaviors such that the association between couple conflict and parenting is stronger for girls compared with boys (Krishnakumar & Buehler, 2000). Conversely, Coiro and Emery (1998) found that mothers compensate for discord in their couple relationship by increasing their closeness to their sons but not their daughters. Furthermore, maternal and paternal depressive symptoms also moderate the link between couple relationship quality and features of the parent-child relationship. For example, Kouros et al. (2014) found that father reports of couple relationship quality (i.e., by using a single item assessing the emotional quality of the spousal relationship) were inversely associated with father-child relationship quality (also reported by fathers) but only in the context of elevated maternal depressive symptoms. To better understand the effects of parental relationship quality on parental talk, it is therefore clear that potential moderating effects, such as parental depression and child gender, should be considered.

## The Current Study

The current study involved 93 families with first-born 7-month-old infants and aimed to examine the association between couple relationship quality, conceptualized as couple conflict, satisfaction, and support, and the frequency of maternal and paternal infant-directed talk. This age group was chosen because before 5 months of age, many adult-infant vocalizations are overlapping, with turn taking becoming more frequent only from 5 months (Gratier et al., 2015; Hilbrink, Gattis, & Levinson, 2015). Using LENA technology enabled us to investigate associations between couple relationship quality and naturalistic language data collected over a full day at home. In particular, once audio information was analyzed using LENA software, both overall frequency of parent speech and frequency of parent-initiated conversational turns with their infant were used to index the home language environment. Given that parental depression is inversely associated with both couple relationship quality and parent-child interactions (e.g., Davis et al., 2011; McLearn et al., 2006; Paulson et al., 2006; Sohr-Preston & Scaramella, 2006) and also moderates the association between couple relationship quality and parent-child interactions (e.g., Kouros et al., 2014), this variable was included as a covariate in our models to isolate the specific association between couple relationship quality and infant-directed speech. In addition, parent and child gender were each examined as moderators of the association between couple relationship quality and the home language environment.

In sum, the current study had three key aims, of which the first was to examine associations between variation in couple relationship quality and (a) overall frequencies of maternal and paternal infant-directed speech and (b) the frequency of mother-/father-initiated conversations with their infant. Accounting for covarying levels of parental depression, the second aim was to examine whether couple relationship quality showed unique associations with either maternal or paternal infant-directed speech. Our third aim was to investigate possible moderating effects of the gender composition of the parent-child dyad on the association between couple relationship quality and infant directed speech. We expected that frequencies of maternal and paternal infant-directed speech would differ across families with high versus low couple relationship quality. Given existing evidence regarding contrasts between mothers and fathers in the relative salience of competing effects of spillover and compensation, we hypothesized that compensation would be clearer for mothers than fathers and might also differ as a function of infant gender.

## Method

### Participants

All participating families (*N* = 93) comprised parents aged 20 years or above, spoke English as their primary language, and were cohabiting with a heterosexual partner. The majority of the participating couples (71/93) were recruited from the New Fathers and Mothers Study, a longitudinal study assessing the transition to parenthood in couples in and around Cambridge, United Kingdom (see Hughes, Lindberg, & Devine, 2018). As well as this sample, 17 couples were recruited from a maternity unit of a local hospital, three couples were recruited from a joint database shared by other researchers at the University of Cambridge, and two couples were recruited via word of mouth. Table 1 summarizes characteristics of participating families. Within this predominantly well-educated sample, parental educational level was unrelated to either the home language environment or to couple relationship quality, *r*s between −.08 and .11, *p*s > .275.[Table-anchor tbl1]

All infants (45 girls, 48%) were healthy and showed typical development at the time of both the collection of information on couple relationship quality when infants were approximately 4 months of age (*M*_age_ = 4.16 months, *SD* = .50, range = 3–6 months, *M*_*boys*_ *=* 4.23 months, *SD* = .59, *M*_*girls*_ = 4.08 months, *SD* = .37) and when the LENA measures were completed at approximately 7 months of age (*M*_age_ = 6.87 months, *SD* = 1.09 months, range = 5–9 months, *M*_*boys*_ *=* 6.94 months, *SD* = 1.04, *M*_*girls*_ = 6.80 months, *SD* = 1.14). The large majority of infants (85%) were between 6 and 8 months when the LENA recording took place.

### Measures

#### Home language environment

The home language environment was recorded using the LENA device (LENA Foundation, 2018), a small recorder inserted into a bespoke vest worn by the infant that recorded up to 16 hr of infant-directed adult words. Compared with most studies that have used the LENA device in typically developing infants, the current sample is relatively large (see Christakis et al., 2009 for an exception, Wang et al., 2017 for a meta-analysis).

Recordings were extracted using the LENA software (for technical reports, see Ford, Baer, Xu, Yapanel, & Gray, 2009) with Advanced Data Extractor (ADEX) software used to conduct additional processing. The average duration of recordings was 15.42 hr (*SD* = 1.47 hr; range = 5.50–16.00 hr). Following procedures used in other studies (e.g., Weisleder & Fernald, 2013), infant naps were excluded from the audio recordings, such that the final sample of recordings had a mean duration of 9.12 hr (*SD* = 1.32 hr, range = 4.51–12.40 hr). The correlation between adult word count before and after correcting for child naps was high, *r*(93) = .92, *p* < .001. ADEX provided information on female and male adult word count and female and male initiated conversational turns (defined as an adult utterance followed by an infant utterance with a maximum of 5 s of silence intervening between). Raw counts of both frequency of adult talk and conversation were converted to hourly rates of talk to control for differences in recording duration. Note that male and female voices were assumed to be those of mothers and fathers; however, these voices could, for some audio segments, refer to other family members present.

LENA has been shown to be reliable for English-speaking families (Oetting, Harfield, & Pruitt, 2009; Oller et al., 2010; Xu, Yapanel, & Gray, 2009). However, as an additional reliability check for the current study, 200 min of audio data were randomly selected and transcribed, allowing the total number of adult words and conversational turns between adult and infant in this period to be counted. These manual transcripts were then compared with the LENA-derived estimates. Results showed that manual counts were very similar to LENA calculations with high intraclass correlations (ICC) for both overall adult word count, ICC = .90 (95% confidence interval = .76–.96) and conversational turn count, ICC = .86 (95% confidence interval = .67–.94).

#### Couple relationship quality

In the extant literature the measurement of couple relationship quality has varied considerably between studies and whereas many studies focus on assessing feelings and evaluations of the couple relationship (e.g., Crohan, 1996; Doss et al., 2009; Pancsofar et al., 2008), others focus on behavioral elements (e.g., Figueiredo et al., 2018; Pratt et al., 1992). To capture the broad nature of couple relationships, we included measures that focus on both behavioral elements (i.e., couple conflict) and emotional evaluations of the relationship (i.e., couple satisfaction and social support).

#### Couple conflict

Items from the Conflict Tactics Scales (CTS) were used to assess intrafamily conflict for participating couples. This questionnaire focuses on verbal aggression and violence, with moderate to high reliability and concurrent and construct validity (Straus, 1979). For the current study, both mothers and fathers completed a shortened seven-item version of the CTS. Each item presented a conflictual scenario (e.g., “My partner or I sulked or refused to talk about an issue”), with a 5-point scale used by each participant to rate its frequency (i.e., *never*, *rarely*, *frequently*, *occasionally*, or *all of the time*) in their couple relationship. Conflict items were all reversed scored such that a higher score indicated less conflict. Mean conflict scores were very similar for mothers, *M* = 23.44, *SD* = 2.16, and fathers, *M* = 23.36, *SD* = 2.37.

#### Couple satisfaction

To assess couple satisfaction, both mothers and fathers completed the 16-item Couple Satisfaction Index (CSI-16), a well-validated measure assessing feelings and evaluations toward a romantic partner (Funk & Rogge, 2007). While including different response scales and formats, each CSI item is designed to measure satisfaction in the participant’s relationship. For example, a set of items requires participants to apply a 6-point scale (ranging from *not at all true* to *completely true*) to indicate their level of agreement with statements regarding their relationship (e.g., “My relationship with my partner makes me happy”). Mean CSI scores were very similar for mothers, *M* = 71.69, *SD* = 8.47, and fathers, *M* = 70.01, *SD* = 10.20.

#### Partner social support

Both mothers and fathers completed the partner subscale of the Multidimensional Scale of Perceived Social Support (MSPSS), which assesses the degree to which couples feel supported by their partner and has good internal and test-retest reliability (Zimet, Dahlem, Zimet, & Farley, 1988). This partner subscale comprised four items (e.g., “There is a special person with whom I can share my joys and sorrows”) rated on a 7-point scale from *strongly agree* to *strongly disagree*. Mean MSPSS scores were very similar for mothers, *M* = 24.91, *SD* = 5.42, and fathers, *M* = 24.97, *SD* = 4.67.

Including items from all three questionnaires of couple relationship quality resulted in high internal consistency for both mothers, Cronbach’s alpha = .79, and fathers, Cronbach’s alpha = .86. Standardized scores for the CTS, CSI, and partner subscale of the MSPSS were therefore summed to provide combined couple relationship quality scores for mothers and for fathers.

#### Depression

Mothers and fathers completed the Centre for Epidemiological Studies Depression Scale (CES-D). The CES-D is a 20-item scale (Radloff, 1977) that indicates the frequency of recent depressive feelings. Items are designed to measure how frequently over the past week the responder has experienced symptoms of depression (e.g., “I was bothered by things that usually do not bother me”), with four possible responses including: *rarely or none of the time (less than 1 day)*, *some or a little of the time (1–2 days)*, *occasionally or a moderate amount of the time (3–4 days)*, and *most or all of the time (5–7 days)*. The CES-D has been found to be a valid and reliable scale to measure depressive symptoms in the general population (Radloff, 1977). Internal consistency in the current study was high, mothers, Cronbach’s alpha = .84, and fathers, Cronbach’s alpha = .90. Using traditional cutoff scores, 11 mothers (12%), and 17 fathers (18%) were above the clinical cutoff for depression in the current study.

### Procedure

As part of a larger framing study (Hughes et al., 2018), couples provided written consent and completed the couple relationship quality and depression scales when the infants were 4 months of age. Approximately 2 months later, the LENA devices were mailed to families with detailed instructions. Parents were requested to use the LENA device to record a typical day when both the mother and father were at home with their infant (e.g., at the weekend). All participating mothers were on maternity leave and at home with their infant. On the day of recording, participants were asked to provide information on any atypical events or activities and to indicate whether additional adults or children were present. If possible, parents were asked to have their infant wearing the device at all times throughout the day. However, if it was necessary to remove the device, as would be the case during bathtime for example, parents were instructed to leave the device nearby so audio information could still be obtained. Ethical approval for this study was approved by the National Health Service Research Ethics Committee.

## Results

First, given that ADEX provides information only on male/female speech, we examined whether both female and male voices were present during the testing day as a way of indirectly examining whether fathers as well as mothers were present on the day of recording. The percentages of 5-min LENA recording segments during which females, males, and both females and males together were heard talking to the infant were calculated. As expected, given mothers were all on maternity leave and at home with their child, female voices were heard speaking to the infant in 92% (*SD* = 5.4%, range = 64–99%) of all 5-min segments. Male voices were heard in 73% (*SD* = 15%; range = 32–96%) of segments, and 71% (*SD* = 15%, range = 32–95%) of all segments were found to contain both female and male speech. These findings are reassuring in that they provide support that both mother and father were present during the whole recording day for the majority of participating families.

Table 2 presents descriptive statistics for the key study variables. Independent-samples *t*-tests revealed no significant difference in total mother or father talk, couple relationship quality (as rated by either mother or father), or on individual parents’ depression scores as a function of infant sex, *t*s < 1.54, *p*s > .128. Compared with fathers, however, mothers spoke more often and initiated more conversations with their infants: paired *t*s > 9.79, *p*s < 0.001.[Table-anchor tbl2]

To examine the first aim of the current study, bivariate associations between mother/father overall word count and conversational turns and couple relationship quality were conducted (see Table 3). Both mother and father report of relationship quality showed a similar negative association with mother word count/initiated conversations. That is, low levels of couple relationship quality (as rated by either mother or father) were associated with higher levels of mother-to-infant overall talk. However, splitting the data set by infant gender showed that these associations were all driven by parents of boys (see Table 3). To assess whether these results remained when accounting for parental depression (Aim 2), partial correlations between couple relationship quality and family talk were conducted, controlling for variation in both mothers’ and fathers’ symptoms of depression. The pattern of findings reported in Table 3 remained unchanged when controlling for parental depression. Note that the frequency of child-initiated conversational turns was unrelated to parental ratings of either couple relationship quality (*r*s < .131, *p*s > .225) or depressive symptoms (*r*s < .159, *p*s > .137).[Table-anchor tbl3]

To complement the correlational analyses described above, we also sought to identify families characterized by low couple relationship quality. To ensure sufficient power, we identified this group as those in the bottom third of the sample for reported couple relationship quality. This categorization identified 29 mothers and 29 fathers as reporting low relationship quality (mother: *M* = −1.16, *SD* = .74; father: *M* = −.91, *SD* = 0.97). These variables were then used in subsequent analyses.

**Figure 1 fig1:**
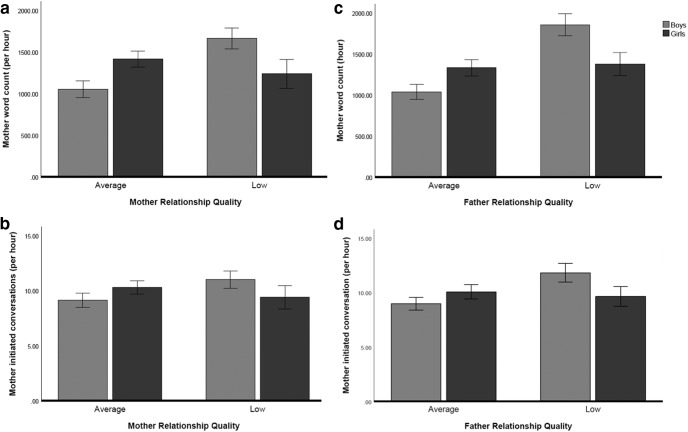
Overall mother talk and mother-initiated conversational turns as a function of both mother (a and b) and father-rated couple relationship quality (c and d) and infant sex.

To examine gender effects (Aim 3), two multivariate analyses of covariance (MANCOVA) with mother/father word count and mother/father-initiated conversations as dependent variables were conducted examining the effect of (a) couple relationship quality (as reported by mother or father, respectively) and infant sex, with depression for both parents included as a covariate. The first 2 (infant sex: boy vs. girl) × 2 (mother relationship quality: low vs. average) MANCOVA on the four talk variables (with mother and father depression entered as covariates) showed no significant main effect for either infant sex or mother relationship quality. However, there was a significant infant sex by mother relationship quality interaction, Λ = .88, *F*(4, 80) = 2.86, *p* = .029. Follow-up separate analyses of covariance showed that this interaction was significant for overall mother word count, *F*(1, 83) = 9.57, *p* = 0.003, and marginally significant for mother-initiated conversations, *F*(1, 83) = 3.10, *p* = 0.081. Thus, the association between mother-reported relationship quality and maternal talk differed for families with sons versus daughters. Specifically, controlling for symptoms of depression in both parents, there was a significant association between mother’s couple relationship quality and mother-infant word count, *F*(1, 43) = 11.26, *p* = .001, partial η^2^ = .207, and a marginally significant association between mother initiated conversation, *F*(1, 43) = 3.30, *p* = .076, partial η^2^ = .07. Specifically, for mothers with sons, low couple relationship quality was associated with greater talk (see Figure 1). However, this association was not found for mothers with daughters, *F*s (1, 39) < 1.01, *p*s > .322.[Fig-anchor fig1]

The second 2 (infant sex: boy vs. girl) × 2 (father relationship quality: low vs. average) on the four family talk variables (with father and mother depression entered as covariates) and showed a significant main effect for father relationship quality, Λ = .86, *F*(4, 77) = 3.11, *p* = .020, which was qualified by a significant infant sex by father relationship quality interaction, Λ = .847, *F*(4, 77) = 3.66, *p* = .009. Follow-up separate analyses of covariance showed that this interaction was significant for mother word count, *F*(1, 80) = 10.71, *p* = 0.002, and mother-initiated conversations, *F*(1, 80) = 4.61, *p* = 0.035. These interactions show that the association of father-reported relationship quality and mothers talk differed for families with sons versus daughters. Follow-up analyses, controlling for both mother and father depression, indicated a significant association between father relationship quality and both mother word count *F*(1, 43) = 21.48, *p* < .001, partial η^2^ = .333, and mother-initiated conversation, *F*(1, 43) = 7.53, *p* = .009, partial η^2^ = .149, for families with boys only, such that low levels of relationship quality was associated with greater talk (see Figure 1); this effect was not found for girls, *F*s(1, 36) < .52, *p*s > 477. All results remained unchanged when controlling for infant age at recording.

## Discussion

The current study examined associations between parental multimeasure ratings of couple relationship quality and the frequency of parent infant-directed talk over the course of a full day. Variation in couple relationship quality is known to be related to developmental outcomes in school-age children (e.g., Brock & Kochanska, 2016; Cummings, Goeke-Morey, & Papp, 2001; Cummings, Davies, & Simpson, 1994) but has been little studied in relation to parent-infant talk. This is a notable omission, given both the established deterioration of the couple relationship at the transition to parenthood (Ahlborg & Strandmark, 2001; Doss et al., 2009) and the importance of parent-infant talk for later development (Rowe, 2008, 2012). Our findings showed that, controlling for both mother and father symptoms of depression, couple relationship quality (as rated by both mothers and fathers) was inversely associated with mothers’ overall word count and mother-initiated conversational turns. However, these effects differed by infant gender: specifically, poor couple relationship quality was associated only with greater talk in mothers with sons.

Compensation may help explain why mothers who are dissatisfied in their couple relationship engage in more talk and initiate more conversations with their infant. Including fathers in the current study enabled us to demonstrate that the compensation hypothesis was specific to mothers. However, further research is needed to establish whether poor couple relationship quality adversely affects other features of parent-infant interactions that are more common in fathers, such as playfulness (Laflamme, Pomerleau, & Malcuit, 2002) rather than talk specifically.

With regard to effects of gender, findings from previous studies involving families with toddlers (e.g., Gilkerson, Richards, & Topping, 2017; Johnson et al., 2014) highlight differences in the amount of talk directed to boys and girls. However, the current study addressed a rather different question, namely whether the magnitude of compensation/spillover varied across parent-infant dyads with contrasting gender composition. In our study, support for a compensatory model was present only among mothers with sons. Specifically, the presence of a fraught or unfulfilling partner relationship was associated with mothers displaying greater investment in their other close male social partner, their son. These findings echo the results from previous research with school-age children in which mothers were found to compensate for discord with their husbands by increasing their closeness to their sons (Coiro & Emery, 1998). Through its focus on infancy, our study extends the developmental scope of gender-specific compensation.

The lack of compensation among mothers with daughters in our study is difficult to interpret, given that our analyses hinged on comparisons across families with first-born infants rather than a within-family design. One possible explanation is that mothers view infant daughters as miniature versions of themselves (rather than of their partners), such that their interactions with daughters are less affected by variation in couple relationship quality. Given the lack of information on the quality or content of parent-initiated talk within this study means that further work is needed to unpack the meaning of the mother-son- mother-daughter–specific findings for talk and conversation. It should be noted that in their longitudinal study across the first year of infancy, Johnson et al. (2014) found greater talk between mothers with infant girls compared with mothers with infant boys. Whereas this finding may seem at odds with the current study’s result that mothers spoke more to boys than girls (in the context of poor couple relationship quality), it is important to note that whereas the current study examined overall mother word count and mother-initiated conversations (which by definition require an infant response to be coded as such), Johnson et al. (2014) focused on the degree to which mothers responded to their infant’s vocalizations. Clearly further research is needed to unpack the nuanced differences in gender-specific parent-child interactions.

Regardless of infant gender, fathers showed significantly less overall talk and initiated fewer conversations than did mothers. Three factors make this contrast particularly striking. First, the data collection period (2016–2017) corresponds to an era of rapidly increasing involvement from fathers in child rearing (Bunning & Pollmann-Schult, 2016; Huerta et al., 2013; Sullivan, Coltrane, McAnnally, & Altintas, 2009). Second, recruitment to the current study hinged on fathers’ willingness to participate in all stages of the research project, and fathers’ high completion rate for the questionnaire-based measures provided clear evidence of their commitment to the study. Third, as a further effort to maximize information on father talk in the home, LENA recordings were requested to take place at the weekend or on a day when the father was home. Interestingly, other recent studies involving parents with infants (e.g., Johnson et al., 2014) have reported similar differences in rates of mother and father talk. Together these findings indicate that fathers’ increased presence does not necessarily equate to increased father-infant interactions. One possible reason may be that despite fathers’ growing involvement in family life, at least in infancy, there remains a striking imbalance between mothers and fathers in responding to basic care needs of their infant (e.g., Laflamme et al., 2002), which represent an opportune time to engage in direct parent-child communication. By including full-day recordings of family talk, it may be that differences in basic care responsibilities between mothers and fathers accumulate to produce substantial differences in frequency of talk and conversation. It should be noted that by virtue of being an audio recording, LENA does not provide information on nonverbal elements of communication, such as eye contact, and certain verbal features, including playful noises, cannot be distinguished from the data. Such elements of communication may be more prevalent in fathers. Therefore, findings are restricted to the information provided by LENA and other potentially important information may be underrepresented by the data.

## Limitations and Future Research

Alongside notable strengths of the study (e.g., relatively large sample size, comprehensive measure of couple relationship quality, and control for depressive symptoms), three limitations deserve note. First, whereas the LENA device provided a unique opportunity to acquire objective and naturalistic data on parent-infant verbal interactions within the home setting and comprised various advantages, including time-saving costs during data collection and coding, the automated software does not provide definitive information on whether the female or male verbalizations were in fact the infant’s mother or father. Furthermore, although LENA provided an insight into parent-infant conversational exchanges, other linguistic features, including the content and tone of interactions, are unknown and may also be susceptible to variability in parent couple relationship quality. These missing features raise an important line of enquiry for future research.

Second, despite the strengths of the study sample in terms of size when compared with other research using LENA (see Wang et al., 2017 for a review), features of the demographic profile of the families limited sample diversity. First, the sample was highly educated, with research demonstrating a relationship between socioeconomic status and child-directed speech (Hart & Risley, 1995; Huttenlocher, Vasilyeva, Waterfall, Vevea, & Hedges, 2007; Hoff, 2003), it is important to further investigate the role of poor relationship quality on parental talk in the context of deprivation as an additional family stressor. Furthermore, the sample comprised only heterosexual and cohabiting parents. Future research with nontraditional families would open up additional avenues for understanding family interactions in the context of poor couple relationship quality and would ensure that these underrepresented family forms are included in research.

Finally, it should be highlighted that the assessments for couple relationship quality took place when infants were 4 months old, whereas the home language environment was collected when infants were approximately 7 months old. Given the rapid developmental changes during the first months of an infant’s life, parents are likely to experience differences in the demands of infant care (e.g., feeding, sleeping, etc.) between 4 and 7 months of age that may impact differentially upon the couple relationship. Further research is needed to better understand short-term temporal changes in couple relationship quality across the first year of an infant’s life and whether this is associated with corresponding changes in infant-directed talk.

As well as providing future directions for further research, the use of recently developed technology to explore parent-child interactions within the current study also highlights potential for real-life applications. In a recent randomized controlled trial, Suskind et al. (2016) devised an intervention to increase the quantity and quality of infant-directed talk, using LENA to provide linguistic feedback. Viewed alongside the current findings, this intervention research highlights the potential of LENA as a means of promoting parent-child talk in the context of psychosocial difficulties, such as couple discord and parental mental health problems.

In conclusion, given that the transition to parenthood is associated with a decrease in couple relationship quality, our findings from this low-risk sample are relatively encouraging. Specifically, variation in couple relationship quality showed no overall association with variation in parent-infant talk. Indeed, for mothers, at least, the experience of poor couple relationship quality may motivate a greater focus on social interactions, at least with their male infants. Future research is needed to examine how well our findings apply to other demographic groups and, based on recent reports that LENA can be used as a means of encouraging parent-infant interactions (Gilkerson et al., 2017; Suskind et al., 2016), understand how best to use LENA at the transition to parenthood to increase father-infant interactions.

## Figures and Tables

**Table 1 tbl1:** Demographic Details for Participating Mothers and Fathers

	Parent characteristics (*n* = 93)	
Variables	Mothers	Fathers
Mean age, years (*SD*)	31.99 (4.43)	33.54 (5.96)
Minimum-maximum age	20–45	21–55
Highest education level (%)		
Lower secondary	1.1	5.4
Upper secondary	2.2	2.2
Foundation degree	21.6	25
Undergraduate degree	36.6	35.9
Postgraduate degree	38.7	31.5
Ethnicity (%)		
Caucasian	91.4	92.5
Asian	2.2	6.5
Black-African	2.2	.00
Other	4.3	1.1

**Table 2 tbl2:** Descriptive Statistics for Parental Word Count, Conversations, Couple Relationship Quality, and Depression

	Total (*N* = 93)		Girls (*n* = 45)		Boys (*n* = 48)	
Variables	Mean (*SD*)	Range	Mean (*SD*)	Range	Mean (*SD*)	Range
						
Mother word count	1,350.67 (588.93)	539–4,153	1,369.65 (491.60)	539–2,763	1,332.88 (672.31)	597–4,153
Father word count	665.90 (382.52)	73–1,748	669.57 (327.20)	115–1,565	662.46 (431.47)	73–1,748
Mother-initiated conversations	10.01 (3.37)	2–20	10.05 (3.04)	4–20	9.98 (3.67)	2–20
Father-initiated conversations	4.27 (2.54)	0–3	4.29 (2.54)	0–13	4.24 (2.57)	1–12
Mother couple relationship quality^a^	00 (1.00)	–2.93 to 1.47	.17 (.90)	−2.31 to 1.45	−.15 (1.07)	−2.93 to 1.47
Father couple relationship quality^a^	00 (1.00)	−3.15 to 1.51	.11 (.99)	−3.15 to 1.51	−.10 (1.01)	−2.76 to 1.34
Mother depression	8.40 (6.27)	0–28	8.60 (6.51)	1–28	8.21 (6.10)	0–23
Father depression	9.12 (8.18)	0–34	9.33 (8.48)	0–29	8.94 (7.98)	0–34
^a^ Couple relationship quality is a standardized score.						

**Table 3 tbl3:** Bivariate Correlations Between Couple Relationship Quality and Parent Talk

Total sample	1	2	3	4	5	6	7	8
1. Mother word count	—	.09	.67**	−.11	−.25*	−.26*	.01	−.05
2. Father word count		—	−.02	.69**	−.10	−.02	.04	−.13
3. Mother-initiated conversations			—	.13	−.13	.09	.11	−.06
4. Father-initiated conversations				—	−.08	.07	.07	−.15
5. Mother relationship quality					—	.81**	−.17	−.19
6. Father relationship quality						—	−.23*	−.31**
7. Mother depression scores							—	.07
8. Father depression scores								—

Separate by infant sex	1	2	3	4	5	6	7	8
1. Mother word count	—	.04	.70**	−.16	.06	.04	−.02	−.14
2. Father word count	.11	—	.03	.78**	.13	.12	−.02	−.20
3. Mother-initiated conversations	.65**	−.05	—	.10	.10	.11	.10	−.02
4. Father-initiated conversations	−.08	.64**	.16	—	.10	.04	.17	−.12
5. Mother relationship quality	−.45**	−.24	−.28	−.22	—	.87**	−.15	−.13
6. Father relationship quality	−.45**	−.11	−.24	.08	.76**	—	−.26	−.29
7. Mother depression scores	.03	.08	.11	−.02	−.21	−.21	—	−.01
8. Father depression scores	.01	−.08	−.10	−.19	−.25	−.34*	.19	—
* *p* < .05. ** *p* < .01, girls above the diagonal in the second panel.
